# False friends: Phagocytes as Trojan horses in microbial brain infections

**DOI:** 10.1371/journal.ppat.1006680

**Published:** 2017-12-14

**Authors:** Felipe H. Santiago-Tirado, Tamara L. Doering

**Affiliations:** Department of Molecular Microbiology, Washington University School of Medicine, St. Louis, Missouri, United States of America; McGill University, CANADA

## Introduction

Humans are constantly exposed to pathogenic microbes. The first line of cellular host defense is composed of “professional” phagocytes, cells that efficiently recognize pathogens, internalize them, and then marshal an array of antimicrobial mechanisms to destroy them. Nevertheless, successful pathogens evade or survive such attack. A particularly subversive strategy is to manipulate normal phagocyte behaviors to benefit the microbe, sometimes even turning the phagocyte from a threat to a safe haven. In this environment, the microbes can multiply while protected from immune surveillance, and in some cases, even travel to the most protected host site, the brain. This gives rise to the Trojan horse analogy: like the wooden horse that carried hidden enemies through the gates into the walled city of Troy, phagocytes carry intracellular microbes through the blood–brain barrier (BBB) into the central nervous system (CNS).

## Immune cells in the brain

Traditionally, the brain has been considered an immune-privileged site because it lacks the normal robust inflammatory responses to antigenic challenges. However, it does have an active immune surveillance system [1] that involves the extravasation of leukocytes, mostly monocytes and lymphocytes, into the meninges and cerebrospinal fluid (CSF). This process follows the same general events that occur in other tissues: rolling of the leukocyte, arrest, crawling, and then transendothelial migration [2]. Because the brain microvascular endothelial cells (BMECs) of the BBB are joined by tight junctions and embedded in a proteinaceous matrix [3], transmigrating leukocytes rarely cross the BBB directly. Instead, they cross into the outer meningeal spaces, where the vasculature is devoid of tight junctions, and from this site they monitor the CSF for the presence of immune signals. Additionally, a recently discovered brain lymphatic system samples the perivascular spaces, bypassing the physical cellular barrier composed of BMECs [4, 5]. Even in healthy individuals, therefore, phagocytes are in close proximity to brain tissue, poised to act upon immune signals.

CNS phagocytes actively respond to signals generated by developmental changes, injury, disease, or infection. Such signals include interferons produced by endothelial cells in response to viral pathogens, chemotactic peptides like N-formyl-methionyl-leucyl-phenylalanine (*f*MLP) generated by bacterial pathogens, and inflammatory cytokines released by epithelial cells in response to fungal pathogens [6]. Microglia, phagocytes that are the only resident immune cells in the brain, also produce cytokines and chemokines to recruit other effector cells to that site. Rapid response to these signals is enabled by the normal presence of phagocytes and lymphocytes in the meninges. However, tight regulation of this response is crucial because adult neurons in the CNS generally do not regenerate; if these cells are damaged by any activities of infiltrating phagocytes, they cannot be replaced, potentially resulting in permanent damage. The BBB helps to limit immune infiltration from the blood, aiding the host to mount an immune response that is robust enough to contain infection yet limited to prevent tissue damage. For the most part, this balance is maintained, and brain infection is prevented or controlled.

## Phagocytes as Trojan horses

### A model for Trojan horse transit into the brain

**Fig 1:**
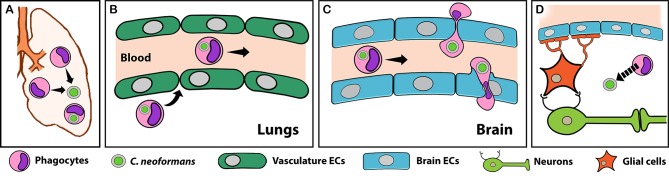
The role of phagocytes as Trojan horses for CNS pathogens. Most neuroinvasive pathogens first infect organs outside the CNS, such as the lungs or the intestines. *Cryptococcus neoformans* infection of the lungs is shown here as an example. Once infection is established, phagocytes (pink cells) are recruited to these sites (A), where they engulf the pathogen. Some infected phagocytes leave the site of infection and enter the bloodstream, facilitated by the highly permeable vasculature (green) of peripheral organs (B). Through a process that is poorly understood, many of these home to the CNS. Once there, infected phagocytes may act as Trojan horses, traversing the BBB (blue cells) with the pathogen as a passenger (C). Although both paracellular (top) and transcellular (bottom) transmigration can occur, the latter is most likely due to the presence of tight junctions in the BBB (C). Once inside the brain, pathogens can potentially exit their Trojan horses and infect other neural structures (D). Parts of this model (A and B) also apply to phagocyte-assisted dissemination and infection outside of the CNS (see text). BBB, blood–brain barrier; CNS, central nervous system; ECs, endothelial cells. Arrows indicate movement; broken arrow indicates fungal egress.

In the absence of trauma, pathogens that cause lethal brain infections (e.g., those in Table 1) reach it from remote sites, generally traveling in the bloodstream. For microbes that use Trojan horse transit, the first step is infection of a phagocyte in the periphery (Fig 1A). Once internalized, the pathogen may actively manipulate the phagocyte to promote migration towards the brain [7]. Alternatively, it may suppress phagocyte activation (and consequent sequestration in the tissue of origin), allowing the infected cell to circulate normally throughout the body (Fig 1B). Once an infected phagocyte reaches the brain, it adheres to the luminal side of brain capillaries (with or without activation of BMECs) and crosses the BBB, either paracellularly (between BMECs) or transcellularly (through BMECs) (Fig 1C). After brain entry, the pathogen may exit its Trojan horse to infect other neural structures (Fig 1D). This model has been elucidated in the most detail for HIV and other viruses [8, 9], but studies reviewed below suggest that similar strategies are used by other microbes that are the focus of this review: bacteria, fungi, and parasites. Aspects of this model may also apply to non-CNS pathogens, such as mucosal pathogens that use phagocytes to disseminate (see “Perspectives and conclusions”).

**Table 1:** Deaths due to CNS infection by select obligate and facultative intracellular microbes.

Pathogen	Growth inside phagocytes	Pathology	Burden (yearly deaths, in thousands)^1^
*Streptococcus pneumoniae*	Yes	Meningitis	113 [35]
*Neisseria meningitidis*	Yes	Meningitis	73.3 [35]
*Mycobacterium tuberculosis*	Yes	Meningitis, encephalitis, intracranial tuberculoma, brain abscesses	55.6 [35]^2^
*Listeria monocytogenes*	Yes	Meningitis, encephalitis, ventriculitis, choroiditis, brain abscesses	0.19 (US) [36]
*Escherichia coli K1*	Yes	Meningitis	Rare
*Cryptococcus neoformans*	Yes	Meningitis, encephalitis, cerebral cryptococcomas	181 [18]
*Histoplasma capsulatum*	Yes	Meningitis, encephalitis	80 [37]^3^
*Coccidioides immitis*	Yes	Meningitis, brain abscesses	<0.20 (US) [38]
*Candida albicans*	Yes^4^	Meningitis, encephalitis	<0.10 (US) [39]^5^
Other fungi (*Aspergillus*, *Mucor*, *Blastomyces*)	Yes^4^	Brain abscesses, meningitis, cerebral stroke	Rare
*Plasmodium falciparum*	No	Brain microvessel obstruction	584 [35]^6^
*Trypanosoma cruzi*	Yes	Meningitis, encephalitis	3.0 [35]^7^
*Toxoplasma gondii*	Yes	Encephalitis	0.20 (US) [39]^8^

^1^ Worldwide deaths as reported in [35] unless denoted (by “US”) as United States burden only.

^2^ This represents 5% of all deaths due to disseminated TB.

^3^ This represents all extrapulmonary infections; a value for deaths due to CNS infection alone is not available.

^4^ These fungi change morphology when inside phagocytes, killing the host cell.

^5^ Most deaths due to invasive candidiasis (approximately 350,000; [37]) are not attributable to CNS infection. This value is based on the US incidence and mortality rate (24%) for CNS candidiasis and the current US population of HIV+ patients.

^6^ This represents 80% of all deaths due to *P*. *falciparum*.

^7^ This represents one-third of all Chagas disease deaths; most are due to heart failure.

^8^ This value is based on the US incidence and mortality rate (14%) for *T*. *gondii* encephalitis and the current US population of HIV+ patients.

Abbreviations: CNS, central nervous system; TB, tuberculosis.

### Bacterial infections

The most common causes of bacterial meningitis are the facultative intracellular pathogens *Streptococcus pneumoniae* and *Neisseria meningitidis*. Because they survive in the blood and can independently interact with BMECs to enter the brain, these microbes do not require Trojan horse transit, although this mechanism may contribute to *S*. *pneumoniae* infection [10]. In contrast, Trojan horses play a central role in infections by another leading cause of bacterial meningitis, *Listeria monocytogenes*.

*L*. *monocytogenes* is a pathogen of humans and domesticated animals that invades the brain parenchyma, unlike most neuroinvasive bacteria, which are limited to the meninges (Table 1). This distinct pathology may relate to its use of Trojan horse invasion, which was first suggested by histological studies showing parasitized phagocytes in the brain tissue of infected mice [11]. Further suggestive of a Trojan horse mechanism were reports that phagocytosis of *L*. *monocytogenes* causes the release of immune signals and activation of BMECs [12], both of which would promote the recruitment of additional leukocytes to the site of infection. More direct support for this mechanism came from the observation that infected mice treated with gentamicin to kill extracellular bacteria still developed CNS infection [13]. This occurred regardless of the initial route of infection, consistent with a general model whereby phagocytes are recruited to the site of infection, engulf the pathogen, and then disseminate (Fig 1). Bolstering this observation, injection of *L*. *monocytogenes*-infected bone marrow myeloid cells caused faster and greater brain colonization than injection of free bacteria [14]. In this study, which used chimeric mice that expressed a fluorescent protein in their bone marrow cells, an increase in fluorescent signal was observed in the brain as the infection progressed, also consistent with a Trojan horse model. The bone marrow and spleen are among the first organs infected by *L*. *monocytogenes*. Interestingly, phagocytes infected in these tissues cannot kill the bacteria but do up-regulate chemokine receptors, making them ideal Trojan horses [15].

### Fungal infections

Fungal infections are responsible for up to 1.6 million deaths every year [3], and the ones affecting the CNS have the highest morbidity and mortality [16]. Although several fungal pathogens cause meningitis (Table 1), the only one to frequently do so is *Cryptococcus neoformans*. Most people have been exposed to this environmental yeast [17]. While healthy individuals are generally asymptomatic, in immunocompromised individuals, the initial pulmonary infection can subsequently disseminate to the CNS. As a result, *C*. *neoformans* is the most common causative agent of meningitis in sub-Saharan Africa and a leading cause of death in HIV+ individuals, killing close to 200,000 people each year [18].

As with *L*. *monocytogenes*, early evidence for Trojan horse transit of *C*. *neoformans* came from histological examination of brains from infected mice [19]. This work was complemented by studies supporting the role of phagocytes in cryptococcal dissemination from the lungs to the brain. For example, depletion of alveolar macrophages reduced dissemination from the lungs [20], systemic monocyte depletion after lung infection reduced fungal burden in other organs [21], and intravenous administration of *C*. *neoformans*-associated macrophages caused higher brain burden than infection with free cryptococci. More recently, direct evidence for Trojan horse transit has come from two studies using *in vitro* models of brain endothelia. Both groups cultured human cerebral microvascular endothelial cell (hCMEC) monolayers on permeable membranes separating the upper (“blood”) and lower (“brain") compartments of tissue culture wells. In one study, a monocytic cell line was first incubated with *C*. *neoformans*, which was engulfed by or adhered to the phagocytes, and the samples were then stained to mark any externally adherent fungi. This mixture was added to the upper chamber, and one day later, monocytes containing unstained fungi were found in the lower chamber, suggesting that Trojan horse crossing had occurred [22]. (Interestingly, the same experiments performed with *Cryptococcus gattii*, a species that primarily causes lung infections, showed less barrier crossing.) In the other study, our group used a flow cytometry strategy to isolate primary human monocytes or macrophages that contained only a single internalized cryptococcal cell. We used this population to directly compare Trojan horse and free fungal transit across a similar BBB model and found that both mechanisms contribute to overall transmigration [23]. We further showed that immune signals that are normally generated during cryptococcal infection preferentially stimulate Trojan horse transit and that this mode of entry provides an alternative for fungal mutants that cannot otherwise traverse the BBB. Finally, we used live microscopy to directly visualize *C*. *neoformans*-infected phagocytes as they crossed model BBB by forming transendothelial pores in the hCMEC. Our microscopic observations also suggested that phagocytes may serve as “taxis” in addition to Trojan horses, contributing to brain infection by picking up the cryptococci (which survive poorly in blood) at distal sites and delivering them to the BBB, where the free fungi can cross independently.

### Parasitic infections

Parasitic infections cause high burdens of disease in low- and middle-income countries, with almost 800,000 deaths in 2015 (Table 1). Several parasites cause devastating CNS pathology, either while remaining in the vasculature—like the parasite that causes malaria—or by crossing the BBB. Here we focus our discussion on a parasite that is estimated to infect one-third of the world, *Toxoplasma gondii* [24].

*T*. *gondii* is acquired orally and colonizes the gastrointestinal tract. In healthy humans, a robust immune response halts the rapid parasite proliferation that would cause severe acute disease in an immunocompromised host. However, even immunocompetent individuals do not completely clear the infection and remain chronically infected with quiescent parasite cysts, mainly in tissues of the CNS and skeletal muscle. Support for Trojan horse transport of *T*. *gondii* derives from studies similar to those mentioned above for other pathogens, mainly adoptive transfer studies showing that parasitized monocytes or dendritic cells cause brain infection faster than free parasites [25]. Consistent with these observations, the injection of intracellular parasites together with antibodies against CD11b, which blocks phagocyte migration, reduced brain burden 2-fold. Furthermore, enhanced transendothelial migration of infected leukocytes has been observed in some, although not all, *in vitro* studies [26, 27]; another study using a robust BBB model consisting of brain endothelia and astrocytes reported the preferential transmigration of infected monocytes [28]. Lastly, *T*. *gondii* Trojan horse transit has been visualized *in vitro*, although these studies used an activated non-brain endothelial cell line (human umbilical vein endothelial cells [HUVECs])[29].

As with the other pathogens discussed here, free *T*. *gondii* likely also cross the BBB, although the relative frequency of the two processes is not known. Notably, infection causes endothelial cells to become activated, with up-regulation of adhesion molecules and down-regulation of junctional complexes [28]; both of these processes could stimulate phagocyte transmigration and thus promote Trojan horse transit. Intravital microscopy has also shown that BMECs serve as a replicative niche for *T*. *gondii* and that intracellular replication is required for egress (through host cell lysis) into the CNS [30]. Interestingly, the same experiments did not show Trojan horse movement, although they did reveal infected phagocytes trapped on the vascular side of brain vessels; these may act as taxis (as with *C*. *neoformans*), serving as a source of free parasites to infect BMECs or cross the BBB. This idea has recently been supported by the observation that adhesion of infected leukocytes to endothelial cells *in vivo* triggers parasite egress [31].

## Perspectives and conclusions

Only a few pathogens cause significant pathology in the brain, yet they collectively lead to over 1 million deaths every year (Table 1). Understanding how these microbes cross the BBB has implications not only for the development of new treatments for these diseases but also for our understanding of the basic immunobiology of the CNS. Here we have presented a general model for Trojan horse infection of the brain and discussed three pathogens that exploit this mechanism. While most of the experimental support for this process comes from *in vitro* studies, new technologies like real-time *in vivo* imaging are beginning to offer exciting insights into this and related processes. Beyond the CNS, bloodstream phagocytes play other critical roles in infection, such as assisting in the dissemination of *Salmonella* from the gut [32], providing a protected replicative niche for *Leishmania* parasites [33], and harboring latent *Mycobacterium* reservoirs [34]. Clearly, understanding the complex interactions between phagocytes and pathogens is of the utmost importance if we wish to elucidate important steps in pathogenesis that can be targeted for efficient control of these deadly infections.
